# Fungicidal Activity and Mechanism of Action of Glabridin from *Glycyrrhiza glabra* L.

**DOI:** 10.3390/ijms222010966

**Published:** 2021-10-11

**Authors:** Anping Li, Zhongmin Zhao, Shaoyong Zhang, Zhijun Zhang, Yanping Shi

**Affiliations:** 1 CAS Key Laboratory of Chemistry of Northwestern Plant Resources, Key Laboratory for Natural Medicine of Gansu Province, Lanzhou Institute of Chemical Physics, Chinese Academy of Sciences (CAS), Lanzhou 730000, China; lianping@licp.cas.cn; 2 Key Laboratory for Quality Control of Chinese Medicinal Materials and Decoction Pieces, Gansu Institute for Drug Control, State Drug Administration, Lanzhou 730000, China; 3 University of Chinese Academy of Sciences, Beijing 100049, China; 4 School of Pharmacy, Lanzhou University, Lanzhou 730000, China; zhaozhm@lzu.edu.cn; 5 Key Laboratory of Vector Biology and Pathogen Control of Zhejiang Province, College of Life Science, Huzhou University, Huzhou 313000, China; 02703@zjhu.edu.cn

**Keywords:** *Glycyrrhiza glabra* L., *Sclerotinia sclerotiorum*, glabridin, fungicidal activity

## Abstract

*Glycyrrhiza glabra* (Licorice) belongs to the Fabaceae family and its extracts have exhibited significant fungicidal activity against phytopathogenic fungi, which has mainly been attributed to the presence of phenolic compounds such as flavonoids, isoflavonoids and chalcones. In this study, a series of licorice flavonoids, isoflavonoids and chalcones were evaluated for their fungicidal activity against phytopathogenic fungi. Among them, glabridin exhibited significant fungicidal activity against ten kinds of phytopathogenic fungi. Notably, glabridin displayed the most active against *Sclerotinia sclerotiorum* with an EC_50_ value of 6.78 µg/mL and was 8-fold more potent than azoxystrobin (EC_50_, 57.39 µg/mL). Moreover, the in vivo bioassay also demonstrated that glabridin could effectively control *S. sclerotiorum*. The mechanism studies revealed that glabridin could induce reactive oxygen species accumulation, the loss of mitochondrial membrane potential and cell membrane destruction through effecting the expression levels of phosphatidylserine decarboxylase that exerted its fungicidal activity. These findings indicated that glabridin exhibited pronounced fungicidal activities against *S. sclerotiorum* and could be served as a potential fungicidal candidate.

## 1. Introduction

Agriculture is the foundation for the development of human society, and the reduction of crop yields by plant diseases is a major obstacle to the sustainable development of agriculture around the world [1,2]. For a long time, chemical fungicides have been the major measure to control crop diseases. Unfortunately, the repeated and extensive use of chemical agents has caused drug-resistance in phytopathogenic fungi, harmed the nontarget organisms, and threated human health and environmental safety [3,4]. These disadvantages have restricted further development of chemical fungicides. Therefore, the quest for highly effective and safe fungicides against plant diseases with novel mode of action is imperative.

Owing to structural diversity, low toxicity and environmental friendliness, bioactive natural products have become important sources of lead compounds to develop biorational alternatives as compared to synthetic fungicides [5,6]. *Glycyrrhiza glabra* L. (licorice) belongs to the Fabaceae family and has been recognized with various pharmacological activities, such as antitussive, expectorant, antiulcer, anti-inflammatory, anticancer and antimicrobial activities [7,8]. Previous studies have demonstrated that the extracts of *G. glabra* exhibited significant fungicidal activity against phytopathogenic fungi, and the pronounced fungicidal activity of the licorice extracts have mainly been attributed to the presence of phenolic compounds such as flavonoids, isoflavonoids, chalcones and bibenzyls [9,10]. However, the identification of active fractions or metabolites, and the mechanism of action against phytopathogenic fungi is unclear.

**Figure 1:**
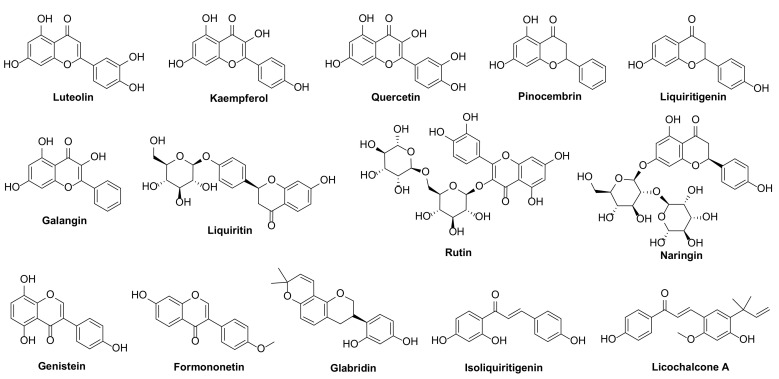
Chemical structures of flavonoids, isoflavonoids and chalcones from *Glycyrrhiza glabra* L.

Therefore, in this study, fourteen phenolic compounds (Figure 1) isolated from *G. glabra*, including flavonoids, isoflavonoids and chalcones, were systematically evaluated for their in vitro fungicidal activity against *Sclerotinia sclerotiorum*, *Botrytis cinerea*, *Fusarium graminearum* and *Rhizoctonia solani*. Moreover, the in vivo fungicidal activity and possible mechanism of the most effective compound against *S. sclerotiorum* were investigated.

## 2. Results and Discussion

### 2.1. In Vitro Fungicidal Activity

The in vitro fungicidal activities of licorice flavonoids, isoflavonoids and chalcones against four phytopathogenic fungi are summarized in Table 1. Among them, all the licorice flavonoids displayed weak fungicidal activity, while the licorice isoflavonoids glabridin was the most active compound against *S. sclerotiorum*, *R. solani*, *F. graminearum* and *B. cinerea* with inhibitory rates of 100%, 100%, 96.21% and 93.12% at 500 µg/mL, respectively. Additionally, licochalcone A also had an excellent antifungal activity against *S. sclerotiorum*, *R. solani* and *B. cinerea*, and the inhibition rates were more than 70%.

The fungicidal spectrum and corresponding EC_50_ values of glabridin were further determined. As shown in Table 2 and Figure S1, glabridin exhibited significant fungicidal activity against all ten plant pathogenic fungi, *B. cinerea*, *S. sclerotiorum*, *F. graminearum*, *M. oryae*, *R. solani*, *Mycosphaerlla melonis*, *Fusarium oxysporum*, *Colletotrichum gloeosporioides*, *Magnaporthe oryzae*, *Thanatephorus cucumeris* and *Phytophthora capsici*, with EC_50_ values ranging from 6.78 to 44.97 μg/mL, which were more potent than those of azoxystrobin. Especially, glabridin showed pronounced fungicidal activity against *S. sclerotiorum*, with an EC_50_ value of 6.78 μg/mL, which was 8-fold more potent than azoxystrobin (EC_50_, 57.39 μg/mL).

### 2.2. In Vivo Fungicidal Activity of Glabridin

Glabridin displayed the best in vitro fungicidal activity against *S. sclerotiorum*, and was then evaluated the in vivo protective and curative effects using detached leaf assay. As shown in Table 3 and Figure S2, the in vivo curative effect of glabridin was stronger than the protective effect. For instance, when the concentrations were 50, 100 and 200 μg/mL, the curative effects reached 33.60%, 64.22% and 81.63%, respectively, while the protective effects were 23.75%, 38.91% and 76.54%, respectively. However, the in vivo fungicidal activity of glabridin was weaker than azoxystrobin. Even so, glabridin still had a great research and application value in agricultural production, since it was easy to degrade, safe for humans and animals, coupled with a good curative effect against *S. sclerotiorum*. Taken together, glabridin showed the excellent in vitro and in vivo fungicidal activity, and effectively controlled plant disease. Hence, it is very meaningful to ulteriorly explore its mechanism of action.

### 2.3. Effect of Glabridin on the Morphology of S. sclerotiorum

**Figure 2:**
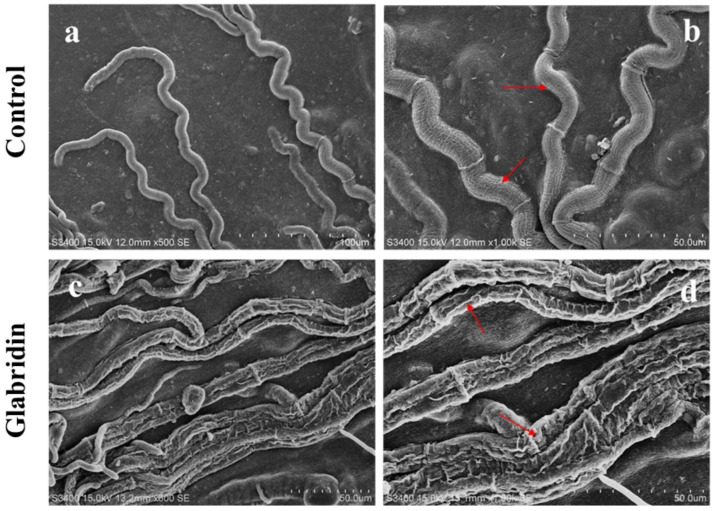
Scanning electron micrographs of *S. sclerotiorum* mycelia in untreated control (**a**,**b**) and treated with 10 μg/mL glabridin (**c**,**d**). Red arrow represents shrunken hyphae. The scale bar in each subfigure: (**a**) 100 μm; (**b**) 50 μm; (**c**) 50 μm; (**d**) 50 μm.

The effect of glabridin on the morphology of *S. sclerotiorum* was observed by both SEM and TEM. As shown in Figure 2, the mycelia of *S. sclerotiorum* with a smooth surface, and an intact structure in the absence of glabridin. Conversely, after treatment with 10 μg/mL glabridin, the mycelia grew abnormally and became distorted. Moreover, the wrinkles and dryness of mycelium morphology were observed.

**Figure 3:**
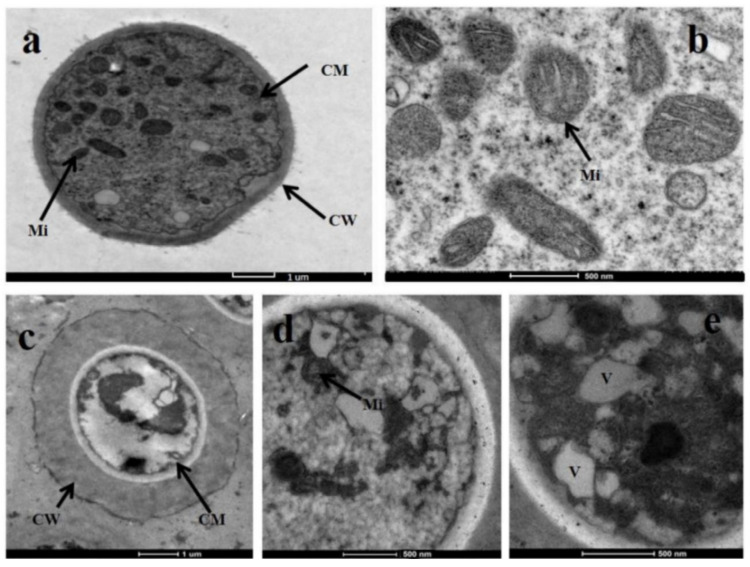
Ultrastructure of *S. sclerotiorum* mycelia by transmission electron microscopy in untreated control (**a**,**b**) and treated with 10 μg/mL glabridin (**c**–**e**). CW: Cell wall; CM: Cell membrane; Mi: mitochondrion; V: vacuole. The scale bar in each subfigure: (**a**) 1 µm; (**b**) 500 nm; (**c**) 1 µm; (**d**) 500 nm; (**e**) 500 nm.

The definitive ultrastructural features of *S. sclerotiorum* mycelia in response to glabridin were observed using TEM. As Figure 3 showed, untreated mycelia had a normal cellular morphology and numerous organelles were observed. In contrast, after exposure to 10 μg/mL glabridin, the organelles became disorganized and caused profound changes. For instance, a significantly thickened cell wall, slightly swollen mitochondria, and an obvious vacuolization were observed. Especially, the cell membranes were invaginated with an obvious plasmolysis.

### 2.4. Effect of Glabridin on the Permeability of Cell Membrane

**Figure 4:**
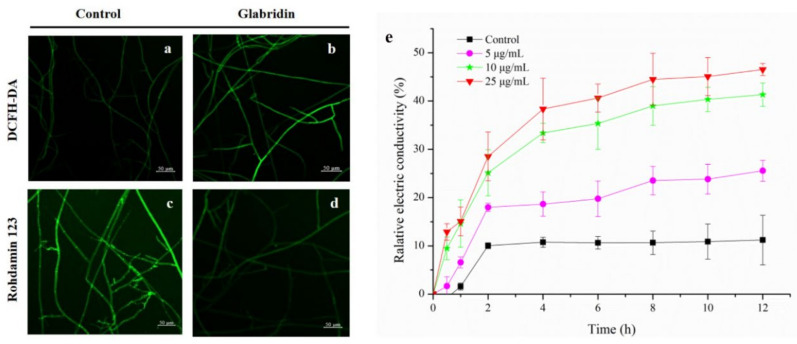
Effects of glabridin on the reactive oxygen species (**a**,**b**), mitochondrial membrane potential (**c**,**d**) and cell membrane permeability (**e**) of *S. sclerotiorum* mycelia. Data are displayed as the mean ± SD. The scale bar: 50 µm.

The severe plasmolysis of *S. sclerotiorum* mycelia treated with glabridin was obviously observed under TEM. Therefore, to confirm whether glabridin acted on the cell membrane of *S. sclerotiorum*, the conductivity changes of the hyphae exposed to glabridin were next evaluated, which could reflect alterations in cell membrane permeability. As shown in Figure 4e, the conductivity of mycelia suspension treated with glabridin increased extremely in a dose- and time-dependent manner compared to that of the control group. This result indicated that glabridin could improve the permeability of cell membranes leading to extracellular electrolyte leakage, resulting in heightened extracellular conductivity which was damaging to the cell membrane of the *S. sclerotiorum* mycelia.

### 2.5. Effect of Glabridin on ROS Production of S. sclerotiorum

ROS can directly damage the cell membrane structure, affect the performance of other functions, and induce cell apoptosis [11]. The accumulation of intracellular ROS might cause the cell membrane permeabilization of *S. sclerotiorum* hyphae [12]. DCFH-DA was used as a ROS indicator to explain the changes of the endogenous ROS production. As shown in Figure 4a,b, compared to the untreated mycelia, the fluorescence intensity of *S. sclerotiorum* mycelia significantly increased after treatment with 10 µg/mL glabridin. The results indicated that glabridin could cause the production and accumulation of endogenous reactive oxygen species in mycelium cells.

### 2.6. Effect of Glabridin on MMP of S. sclerotiorum

The endogenous ROS are primarily generated in the mitochondria, and ROS accumulation could be caused by a change in mitochondrial membrane potential (MMP) [12]. Excess ROS can destroy intracellular macromolecules, decrease mitochondrial membrane potential, disrupt mitochondrial membrane permeability, release cytochrome C, and as a result, induce cell apoptosis [13]. The effect of glabridin on MMP was subsequently assessed using rhodamine 123. The results presented in Figure 4c,d showed that the fluorescence intensity and density of the glabridin-treated mycelial were significantly weakened compared to that of the control group, which supported the evidence of the capacity of glabridin to potently compromise mitochondrial function by interfering with the membrane potential of normal mitochondria, thereby arresting the growth of cells.

### 2.7. Transcriptomic Analysis

#### 2.7.1. RNA- Sequencing, Assembly and Annotation

Omics tools has been widely used to understand the molecular mechanisms of compounds against pathogens [14]. Thus, we conducted transcriptomics analyses to explore the mode of action of glabridin. As illustrated in Table 4, two cDNA libraries, CO (Control hyphae) and TR (Treated hyphae) were sequenced by Illumina Hiseq 4000, yielding 145.49 and 141.99 Mb Raw reads, respectively. After assembling and eliminating of redundant data, we obtained 131.91 Mb clean reads from CO and 128.65 Mb from TO, and the total amounts of nucleobases obtained from the clean reads were 19.78 and 19.29 Gb, respectively. The gene expression of CO and TR groups were then analyzed. As shown in Figure S3, compared with control group, a total of 720 differentially expressed genes (DEGs) were regulated in the glabridin-treated mycelia. Among them, 383 genes were up-regulated and 337 genes were down-regulated.

#### 2.7.2. Gene Ontology (GO) Functional Analysis of DEGs

**Figure 5:**
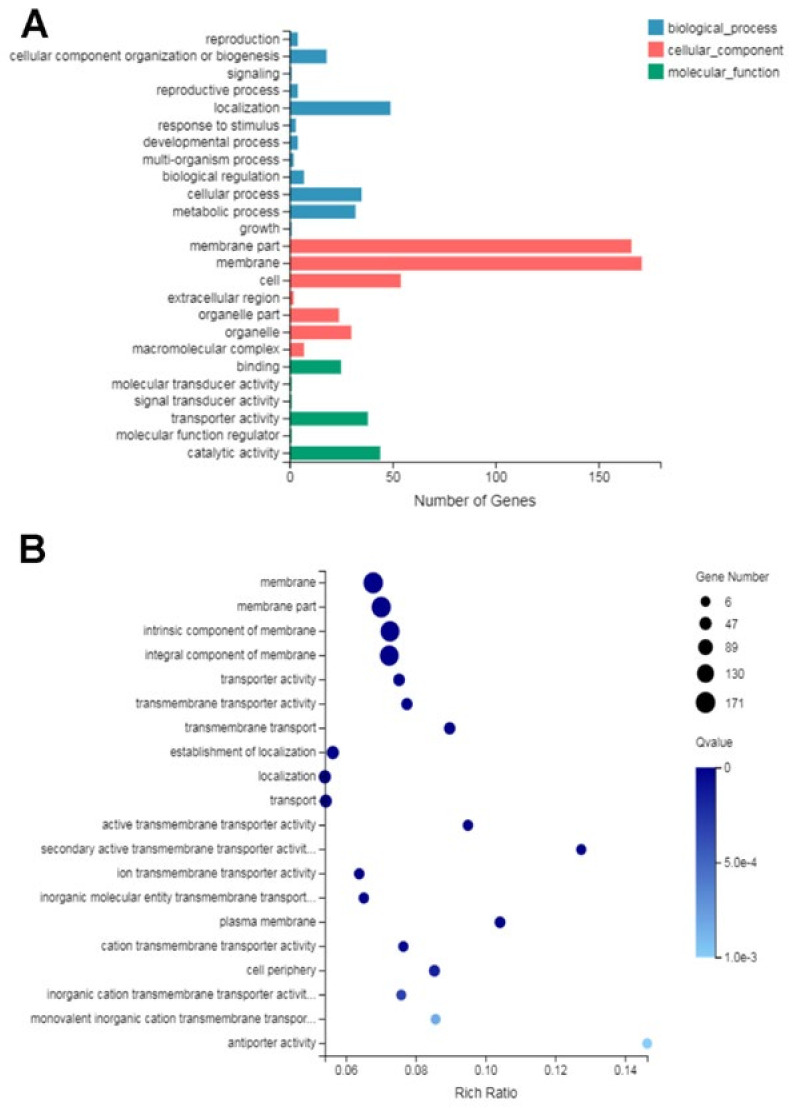
GO analysis of DEGs. (**A**) GO classification of DEGs. (**B**) GO annotation of DEGs related to membrane and membrane components.

In this study, GO functional classification was performed on 720 DEGs. As shown in Figure 5, in the biological process ontology, most of DEGs were classified into cellular process, metabolic process and localization; in the cellular component ontology, most of DEGs were related to the cell, membrane part, membrane and organelle; in the molecular function ontology, most of DEGs were associated with catalytic activity, transporter activity and binding. Moreover, cellular component is the largest in the entire GO functional annotation, so we speculated that the mode of action of glabridin may be related to this part, which was consistent with the result of TEM observation and the cell membrane permeability.

#### 2.7.3. KEGG Functional Analysis of DEGs

**Figure 6:**
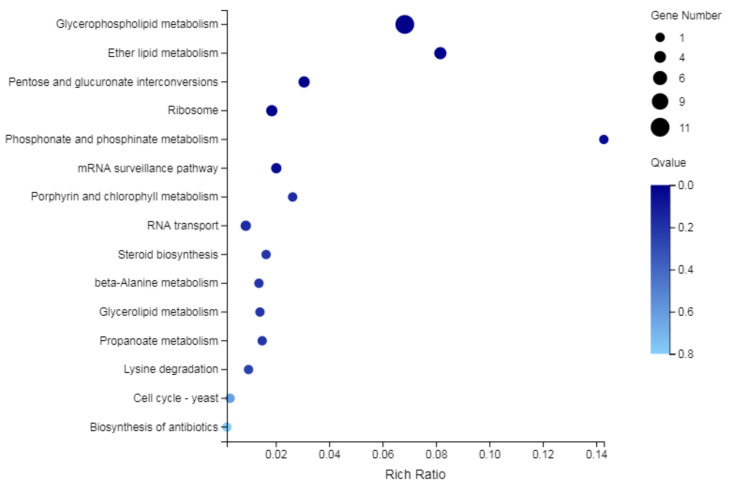
KEGG analysis of DEGs related to membrane and membrane components.

Based on GO functional annotation, the KEGG pathway analysis of the DEGs involved membrane and membrane components were carried out. As shown in Figure 6, the DEGs were mainly enriched in the glycerophospholipid metabolism pathway and refered to 11 DEGs in total, of which 5 DEGs (SSIG_14322, SSIG_12051, SSIG_05104, SSIG_04079and SSIG_04360) are related to phosphatidylserine decarboxylase (PSD) (Table 5). These results indicated that glabridin might act on phosphatidylserine decarboxylase that exerted its fungicidal activity.

### 2.8. RT-qPCR Verification

**Figure 7:**
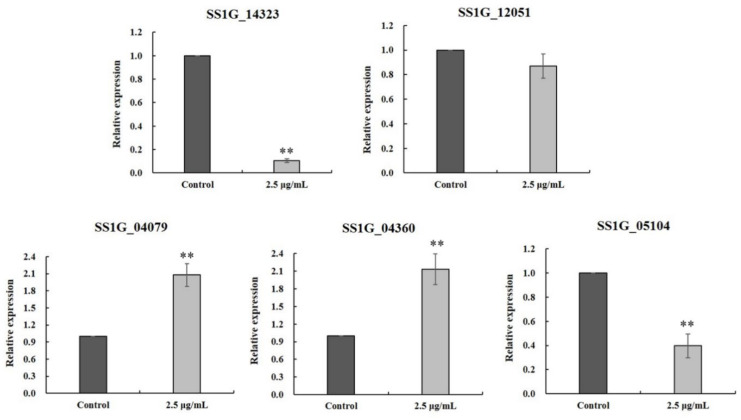
Verification of DEGs by RT-qPCR. Data are displayed as the mean ± SD, bars with the ** indicate very significant differences according to Duncan’s test (*p* < 0.01).

Phosphatidylserine decarboxylases (PSD) regulate phosphatidylethanolamine biosynthesis, and are important for mycelial growth, sexual and asexual reproduction, virulence in phytopathogenic fungi [15]. The results of transcriptomic analysis showed that glabridin might act on phosphatidylserine decarboxylase located in the mitochondria intima. Therefore, the real-time qPCR was performed to verify the expression levels of five key DEGs related to the regulation of phosphatidylserine decarboxylase. As shown in Figure 7, the relative expressions of SSIG_14322, SSIG_12051 and SSIG_05104 were down-regulated, while the relative expressions of SSIG_04079 and SSIG_04360 were up-regulated, which was consistent with the results of the transcriptomic analysis.

### 2.9. Effect of Glabridin on Sclerotia Formation and Germination

Sclerotia is the hypopus of *S. sclerotiorum*, which can survive for a long time under harsh conditions the primary and become the primary infection sources to infect rape plant [16]. As shown in Figure S4, glabridin significantly inhibited the sclerotia formation of *S. sclerotiorum* with a concentration-dependent manner. The inhibition rates of sclerotia formation reached 43.3%, 62.6%, and 66.9 % at 10, 25 and 50 µg/mL, respectively, which were slightly higher than broad-spectrum fungicide azoxystrobin (34.8%, 43.1%, and 62.1%, respectively). Moreover, glabridin also could inhibit the sclerotia germination of *S. sclerotiorum* with the germination inhibitory rates of 36.7% and 63.3% at 25 and 50 µg/mL, respectively, and were more potent than azoxystrobin. Collectively, these results demonstrated that glabridin could inhibit sclerotium formation and germination of *S. sclerotiorum*, thereby reducing the primary infection sources and controlling the disease.

## 3. Materials and Methods

### 3.1. Pathogenic Fungi and Reagents

The tested strains, *Sclerotinia sclerotiorum*, *Mycosphaerlla melonis*, *Botrytis cinerea*, *Fusarium graminearum*, *Fusarium oxysporum* f. sp. Vasinfectum, *Colletotrichum gloeosporioides* and *Magnaporthe oryzae* were provided by Institute of Plant Protection, Gansu Academy of Agricultural Science; *Thanatephorus cucumeris*, *Phytophthora capsici* and *Rhizoctonia solani* were obtained from Pesticide Application Laboratory, Environment and Plant Protection Institute of Chinese Academy of Tropical Agricultural Science. All strains were clinically isolated and incubated in potato dextrose agar (PDA) at 25 °C to get new mycelia for the fungicidal test.

The licorice flavonoid, isoflavonoid and chalcone compounds were purchased from Chunqiu Biological Engineering Co., Ltd. (Nanjing, China), and the chemical structures are shown in Figure 1. Azoxystrobin was supplied by Shanggezhilu Bio-Technology Co., Ltd. (Xi’an, China) and used as a reference fungicide.

### 3.2. In Vitro Antifungal Assay

The in vitro fungicidal activity of tested compounds against *S. sclerotiorum*, *B. cinerea*, *F. graminearum* and *R. solani* was evaluated using mycelium growth rate method as described previously [17]. Briefly, the compounds were dissolved in dimethyl sulfoxide (DMSO), and then mixed with sterile molten PDA to obtain the final concentrations ranging from 5 to 500 μg/mL. The plugs of mycelia (5 mm in diameter) of phytopathogenic fungi were inoculated on PDA plates and then were incubated at 25 °C in the dark. All the tests were triplicated. The blank control was maintained with 0.5% DMSO (*v*/*v*) mixed with PDA and azoxystrobin was used as a positive control. The diameters (mm) of inhibition zones were measured by the cross-bracketing method, and the growth inhibition rates were calculated when the blank control hyphae grew to the edge of the petri dish according to the following formula:

Mycelial growth inhibition (%) = [(dc − dt)/(dc − 5 mm)] × 100

where dc and dt are average diameters of the fungal colony of control and treatment, respectively.

### 3.3. In Vivo Fungicidal Assay

The protective effect and curative effect of glabridin against S. sclerotiorum in leaves of rape were performed according to previously described methods [16]. Five-leaf-aged rape (LongYou 5#) were harvested from greenhouse of Gansu Academy of Agricultural Science, China. Firstly, the rape leaves were washed three times with distilled water and sterilized by immersion in 70% ethanol for 1 min. For protective effect assay, the leaves were sprayed with different concentrations of glabridin (50, 100, and 200 µg/mL) aqueous solution comprised 0.1% Tween 80 and 1% DMSO (*v*/*v*). After 24 h, the fungus cakes were inoculated on the leaves. Azoxystrobin with different concentrations (25, 50, and 100 µg/mL) served as the positive control. All treatments were five replicates. Then, the inoculated leaves were cultured three days in an artificial climate incubator with a photoperiod of 16 h, relative humidity of 85%, and a temperature of 21 ± 1 °C. For curative effect assay, mycelial plugs were inoculated on the leaves, and after 24 h inoculation the leaves were sprayed with glabridin in same concentrations. Finally, the diameter of leaf lesions were measured, and protective effect and curative effect (%) were calculated according to the following formula:

Ec (Ep) (%) = 100 × (Db − Dt)/Db

where Ec represents the protective effect, Ep represents the therapeutic effect, Db represents the average diameter of the control leaves, and Dt represents the average diameter of the treatment group.

### 3.4. Transcriptomics Analysis

*S. sclerotiorum* mycelia (5 mm) were placed in 60 mL PD medium and shaken at 140 rpm for 24 h at 25 °C in the incubator, and then glabridin was added to the medium at a final concentration of 5 μg/mL. After incubation for 3 days, the mycelia were harvested, washed and stored in liquid nitrogen. The mycelia grown without glabridin was used as a control.

The total RNA of *S. sclerotiorum* was extracted with the fungal RNA extraction kit (Omega, Norcross, GA, USA) according to the user manual. The concentration and quantity of RNA were determined using a Nanodrop system (NanoDrop, Madison, Wilmington, NC, USA) and an Agilent 2100 Bioanalyzer (Agilent RNA 6000 Nano Kit, Agilent, Santa Clara, CA, USA), respectively. Then, the construction and sequencing reaction of cDNA library were performed by Beijing Genomics Institute Co., Ltd. (Beijing, China). In brief, the total RNA was purified using Oligo-dT magnetic beads, and then sheared it into short fragments with fragmentation buffer. Afterwards, the first strand of cDNA was synthesized with random N6 primers, and the second strand of cDNA was acquired using DNA polymerase and RNase. Then, the terminal of ds-cDNA were repaired, the A-base were added, and Illumina adapters were ligated to the cDNA fragments. The fragment size was selected with AMPure XP beads, and the ligation products were amplified by PCR and purified again with AMPure XP beads. After that the range of inserted fragments and the concentration of the library were checked with Agilent 2100 Bioanalyzer and ABI StepOnePlus Real Time PCR System (TaqMan Probe), respectively. Finally, the cDNA libraries were sequenced by using Illumina HiSeqTM 4000 (Illumina, San Diego, CA, USA). Differential expression analysis of treatment group and control group was performed using DEGseq2. The significant levels of terms and pathways were corrected by q value. A threshold of the *p* < 0.05 and log2-fold change >1 were considered to be significantly differentially expressed [18]. Differentially expressed genes were selected and analyzed further.

### 3.5. RT-qPCR Verification

The total RNA of *S. sclerotiorum* mycelia was extracted, and the RNA concentration was determined using an ultramicro spectrophotometer (Thermo, Waltham, MA, USA). Then the total DNA-free RNA was regarded as a template for cDNA synthesis using the FastKing gDNA Dispelling RT SupperMix reverse transcription kit (Tiangen, Beijing, China), and the synthesized cDNA was stored at −20 °C. Five gene sequences were obtained from GenBank, and the primers were designed with Primer 3 software (Table 5). The real-time qPCR was performed with SuperReal PreMix Plus (Tiangen, China), using the QuantStudio 5 real-time PCR systems (Thermo, Waltham, MA, USA). Finally, the RT-qPCR was run using the following program: 95 °C denaturation for 15 min and 40 cycles of 95 °C denaturation for 15 s, 55 °C annealing for 30 s and 72 °C extension for 32 s. The relative expression of each gene was calculated by 2^−ΔΔCT^ method [19], and each treatment was replicated three times.

### 3.6. Scanning Electron Microscopy (SEM) Observations

The changes of glabridin on *S. sclerotiorum* microstructure were observed using scanning electron microscopy (SEM) according to the method described in our previous study [17]. After treated with 10 μg/mL glabridin, the mycelial disks (5.0 × 4.0 mm) were cut from the growth boundary of the fungi on PDA and fixed in 4% glutaraldehyde solution for one day at 4 °C. Then, the disks were washed with 0.01M PBS and fixed with 1% osmium tetraoxide solution (*w*/*v*) for 2 h. The samples were washed with 0.01M PBS and dehydrated in a graded ethanol series. Finally, after drying at a critical point and gold-sprayed, the samples were observed using SEM at an accelerating voltage of 10 kV.

### 3.7. Transmission Electron Microscopy (TEM) Observations

TEM observations on the hyphae of *S. sclerotiorum* were performed according to the described method with some modifications [20]. The dehydrated mycelial blocks were cut into thin sections and then double-stained with uranyl acetate and lead citrate after being embedded in resin, and the samples were observed with a Tecnai transmission electron microscope at 120 kV.

### 3.8. Effect on Cell Membrane Permeability

The conductivity changes of *S. sclerotiorum* mycelial exposed to glabridin were evaluated to reflect alterations in cell membrane permeability according to our previously reported method [17].

### 3.9. Effect on the Reactive Oxygen Species (ROS)

The accumulation of reactive oxygen species was measured according to the previously described method [21]. In a short, the 5 mm *S. sclerotiorum* mycelia tips treated with glabridin at 10 μg/mL were incubated on a sterile slide for 72 h at 25 °C. The mycelium were then stained with 10 μM 2′,7′-dichlorodihy drofluorescein diacetate (DCFH-DA) solution and incubated for 20 min at 37 °C in the darkness. After incubation, the stain was carefully removed and the mycelium were washed three times with pre-cooled 0.01 M PBS. The samples were observed using a LSM 800 laser confocal microscope.

### 3.10. Effect on the Mitochondrial Membrane Potential (MMP)

The effect of glabridin on the MMP of *S. sclerotiorum* mycelia was evaluated according to the method described in our previous study [17,21]. The hyphae treated with 10 μg/mL glabridin were stained with 2 μM Rhodamine 123 solution and incubated for 30 min at 37 °C in the darkness. After incubation, the stain was carefully removed and the mycelium were washed three times with 0.01 M PBS. The samples were observed using a LSM 800 laser confocal microscope.

### 3.11. Effects on Sclerotia Formation and Germination

The effects of glabridin on the *S. sclerotiorum* sclerotia formation and germination were evaluated according to the described method [22]. For sclerotia formation inhibition assay, the PDA plates treated with different concentrations of glabridin (10, 25 and 50 μg/mL) were prepared and inoculated *S. sclerotiorum* cakes (5 mm) on the center of the plate. Azoxystrobin served as the positive control and each concentration is repeated three times. The samples were incubated 15 days at 25 °C in the dark. The sclerotia formed were collected, dried at 60 °C for 24 h, and then weighed. The inhibitory rate of sclerotia formation was calculated.

For sclerotinia germination inhibition assay, the sclerotia of *S. sclerotiorum* were obtained according to the above method. Firstly, the PDA medium containing various concentrations of glabridin were prepared, and then sclerotia were placed on the culture. Azoxystrobin served as a positive control, and each treatment consisted of three replicates. All of the treatments were incubated at 25 °C for 24 h, and then the inhibitory rate was calculated.

### 3.12. Statistical Analysis

The fungicidal assay was performed with three biological replicates, at least two independent experiments for the tested compounds, in order to confirm the activity. The results were presented as the mean ± SD. To analyze the differences, one-way analysis of variance (ANOVA) was performed using SPSS 24.0 and Duncan’s statistical procedure was utilized to compare the means.

## 4. Conclusions

In summary, a series of licorice flavonoids, isoflavonoids and chalcones were evaluated for their fungicidal activity against phytopathogenic fungi. Of these compounds, glabridin exhibited significant fungicidal activity against ten kinds of phytopathogenic fungi. Importantly, glabridin was observed as the most active against *S. sclerotiorum*, and was more potent than azoxystrobin. Moreover, glabridin exhibited excellent in vivo protective and curative activities against *S. sclerotiorum*. The preliminary mechanistic study showed that glabridin could cause ROS accumulation, the loss of mitochondrial membrane potential and cell membrane destruction through effecting the expression levels of phosphatidylserine decarboxylase. Further studies on a detailed mechanism are in progress.

## Figures and Tables

**Table 1:** In vitro fungicidal activity of compounds from *Glycyrrhiza glabra* at 500 µg/mL after three treatments.

Flavonoids	Inhibition Rate (%) ± SD			
	*S. sclerotiorum*	*R. solani*	*F. graminearum*	*B. cinerea*
Luteolin	35.20 ± 0.70	NA	4.63 ± 0.34	35.08 ± 1.69
Kaempferol	NA	26.24 ± 1.18	2.97 ± 0.37	16.02 ± 0.67
Quercetin	12.56 ± 0.35	NA	14.37 ± 0.29	32.78 ± 1.56
Pinocembrin	18.14 ± 1.25	51.88 ± 0.60	18.93 ± 1.54	31.65 ± 0.48
Liquiritigenin	30.02 ± 0.57	30.50 ± 0.41	53.26 ± 0.71	36.48 ± 1.57
Galangin	NA	10.90 ± 0.95	20.40 ± 1.20	13.92 ± 0.80
Liquiritin	1.91 ± 0.40	5.58 ± 0.68	2.39 ± 0.60	3.00 ± 0.54
Rutin	24.42 ± 0.32	22.80 ± 0.51	24.38 ± 0.43	37.79 ± 0.61
Naringin	7.59 ± 0.98	9.68 ± 1.17	2.34 ± 0.82	11.62 ± 0.98
Genistein	4.30 ± 0.69	4.30 ± 0.69	34.25 ± 1.48	21.10 ± 0.52
Formononetin	NA	2.87 ± 0.45	6.28 ± 0.80	6.63 ± 0.69
Glabridin	100 ± 0.00	100 ± 0.00	96.21 ± 0.76	93.12 ± 0.81
Isoliquiritigenin	31.71 ± 0.70	34.15 ± 0.85	55.29 ± 0.58	51.38 ± 0.44
Licochalcone A	76.10 ± 0.54	80.19 ± 0.54	18.58 ± 0.52	73.28 ± 0.64
Azoxystrobin	97.13 ± 0.47	100 ± 0.00	100 ± 0.00	100 ± 0.00

Data are displayed as mean ± SD. NA = no activity.

**Table 2:** In vitro fungicidal activity of glabridin against ten plant pathogenic fungi.

Compounds	Pathogenic Fungi	Virulence Equation (Y = ax + b)	Correlation Coefficient (R2)	EC50 (µg/mL)
Glabrindin	*B. cinerea*	y = 0.6094x + 4.4290	0.8672	8.65
	*S. sclerotiorum*	y = 2.2922x + 2.7926	0.9295	6.78
	*F. graminearum*	y = 1.0731x + 3.6297	0.9452	18.92
	*M. oryae*	y = 1.4210x + 3.2215	0.8943	17.85
	*F. oxysporum*	y = 0.6476x + 4.4204	0.9833	9.85
	*R. solani*	y = 2.1669x + 2.4477	0.9699	15.06
	*M. melonis*	y = 1.3578x + 3.4252	0.9717	14.45
	*P. capsici*	y = 1.9803x + 2.1392	0.9845	27.84
	*T. cucumeris*	y = 0.4647x − 1.0538	0.8924	8.33
	*C. gloeosporioides*	y = 0.4889x − 0.7973	0.9545	44.97
Azoxystrobin	*B. cinerea*	-	-	>50
	*S. sclerotiorum*	-	-	>50
	*F. graminearum*	y = 2.0532x − 8.8182	0.8882	27.43
	*M. oryae*	y = 0.9405x − 3.6062	0.9641	12.04
	*F. oxysporum*	y = 2.3424x − 10.265	0.9946	27.96
	*R. solani*	y =1.4638x − 5.6362	0.9400	49.29
	*M. melonis*	y = 2.0431x − 8.9002	0.9744	20.40
	*P. capsici*	-	-	>50
	*T. cucumeris*	y = 0.571x + 4.1224	0.9347	34.43
	*C.gloeosporioides*	-	-	>50

**Table 3:** In vivo fungicidal activity of glabridin against *S. sclerotiorum*.

Compounds	Concentration (μg/mL)	Protective Effect		Curative Effect	
		Lesion Length (mm ± SD)	Control Efficacy (%)	Lesion Length (mm ± SD)	Control Efficacy (%)
Glabridin	50	13.70 ± 0.96	23.75	17.65 ± 1.14	33.60
	100	11.20 ± 0.89	38.91	11.82 ± 0.99	64.22
	200	9.30 ± 1.01	76.54	8.50 ± 1.07	81.63
Azoxystrobin	25	8.10 ± 0.75	71.82	7.82 ± 0.42	85.21
	50	7.00 ± 0.08	81.40	6.58 ± 0.44	91.69
	100	6.00 ± 0.10	90.13	6.28 ± 0.19	93.26
Control	-	17.65 ± 1.70	-	24.05 ± 1.13	-

**Table 4:** Summary of sequence analysis.

Sample	Raw Reads (Mb)	Clean Reads (Mb)	Clean Bases (Gb)	Q20 (%)	Q30 (%)	Clean Reads Ratio (%)
CO1	49.08	44.1	6.61	97.19	89.34	89.84
CO2	49.08	44.65	6.7	97.28	89.6	90.97
CO3	47.33	43.16	6.47	97.51	90.11	91.19
TR1	47.33	43.21	6.48	97.14	89.15	91.3
TR2	47.33	42.81	6.42	97.22	89.44	90.46
TR3	47.33	42.63	6.39	97.12	89.14	90.08
CO summary	145.49	131.91	19.78	97.33	89.68	90.67
TR summary	141.99	128.65	19.29	97.16	89.24	90.61
Summary	287.48	260.56	39.07	97.25	89.46	90.64

**Table 5:** Key DEGs involved in phosphatidylserine decarboxylase and their primer sequences in qRT-PCR.

Gene Symbol	FPKM (Control)	FPKM (Treat)	Log2FC	Annotation	Primer Sequences
SSIG_14322	23.42	7.45	−1.66	phosphatidylserine decarboxylase	F- GTGGTGGGAGTGATCCTTATATC R- GAGTGACAAGCAAAGCACAAG
SSIG_12051	4.06	1.91	−1.04	phosphatidylserine decarboxylase	F- CGGATTCACCACGACGATAAT R- GACTTCCACATGGACTCGTAAG
SSIG_05104	1.86	5.84	1.65	phosphatidylserine decarboxylase	F- AGCAAATCAGGCTGGAGATAG R- GTCTGAGTAATAACCGTCGTCTT
SSIG_04079	8.86	18.70	1.12	phosphatidylserine decarboxylase	F- AGCAAATCAGGCTGGAGATAG R- GTCTGAGTAATAACCGTCGTCTT
SSIG_04360	6.13	3.19	−1.16	phosphatidylserine decarboxylase	F- CCGCTGTCATCAGAACCTATT R- CTACTGCGCACCATACGATAA

## Data Availability

All data are contained within this manuscript.

## Supplementary Materials

The following are available online at https://www.mdpi.com/article/10.3390/ijms222010966/s1.
