# Supplementary material for: Fungicidal Activity and Mechanism of Action of Glabridin from Glycyrrhiza glabra L
Source: Int J Mol Sci. 2021 Oct 11;22(20):10966. doi: 10.3390/ijms222010966 (PMC8537655; doi:10.3390/ijms222010966)
Supplement: Supplementary file 1 [file ijms-22-10966-s001.zip › ijms-1402314-supplementary.pdf]

Supporting Information

Fungicidal activity and mechanism of action of glabridin from *Glycyrrhiza glabra* L.

An-Ping Li [a,b,e], Zhong-Min Zhao [c,d], Shao-Yong Zhang [c], Zhi-Jun Zhang [d*], Yan-Ping Shi [a*]

[a] CAS Key Laboratory of Chemistry of Northwestern Plant Resources and Key Laboratory for Natural Medicine of Gansu Province, Lanzhou Institute of Chemical Physics, Chinese Academy of Sciences, Lanzhou 730000, P. R. China

[b] Gansu Institute for Drug Control, Key Laboratory for Quality Control of Chinese Medicinal Materials and Decoction Pieces, State Drug Administration , Lanzhou 730000, P. R. China

[c] Key Laboratory of Vector Biology and Pathogen Control of Zhejiang Province, College of Life Science, Huzhou University, Huzhou 313000, China;

[d] School of Pharmacy, Lanzhou University, Lanzhou 730000, People's Republic of China;

[e] University of Chinese Academy of Sciences, Beijing 100049, P. R. China

[*] To whom correspondence should be addressed.

zhangzhijun198803@163.com (Z.J. Zhang)
shiyp@licp.cas.cn (Y.P. Shi).

Supplementary contents

Figure S1 *In vitro* antifungal activities of glabridin against *S. sclerotiorum.*

Figure S2 *In vivo* curative and protective effects of glabridin against S. sclerotiorum.

Figure S3 The numbers and MA distribution of DEGs.

Figure S4 Effect of glabridin on sclerotia formation and germination of S. sclerotiorum.

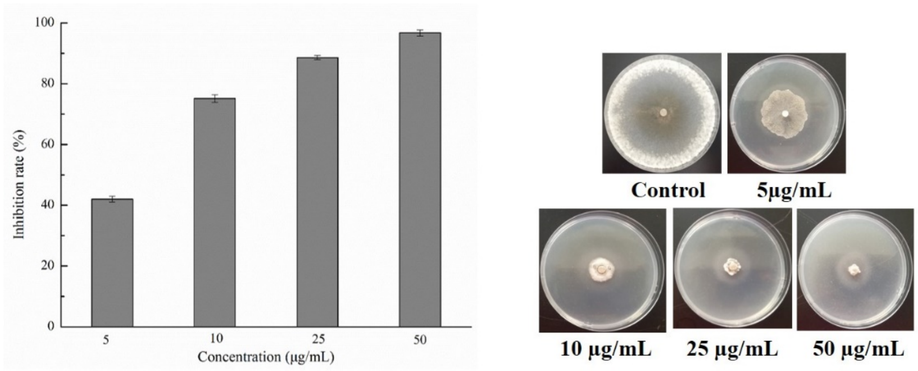

Figure S1 *In vitro* antifungal activities of glabridin against *S. sclerotiorum.*

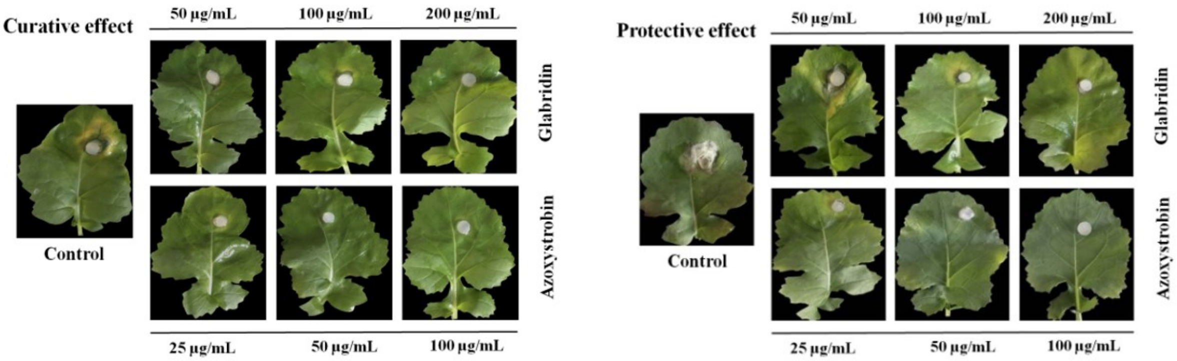

Figure S2 *In vivo* curative and protective effects of glabridin against S. sclerotiorum.

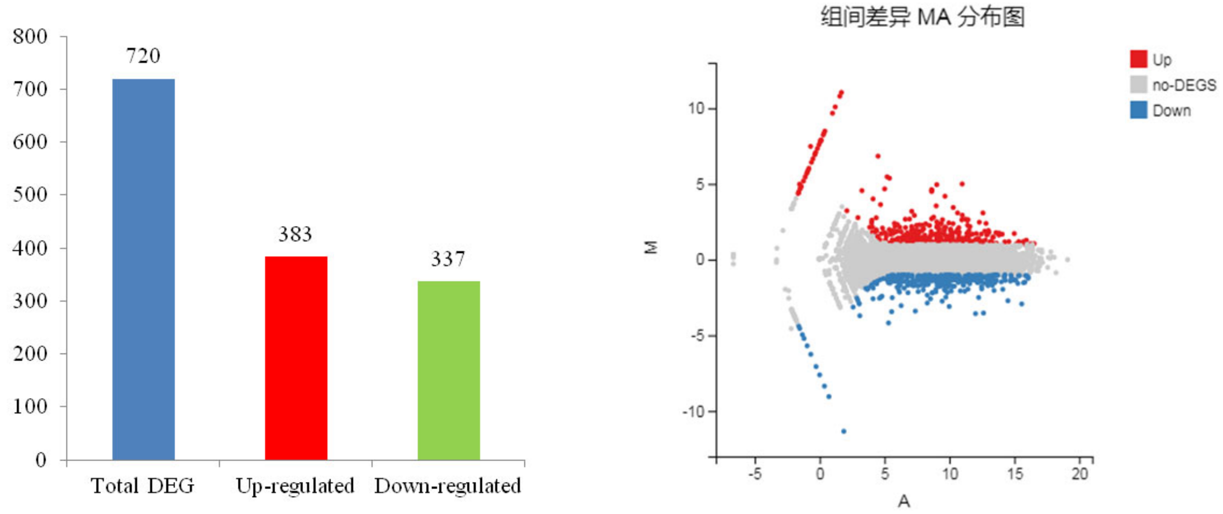

Figure S3 The numbers and MA distribution of DEGs.

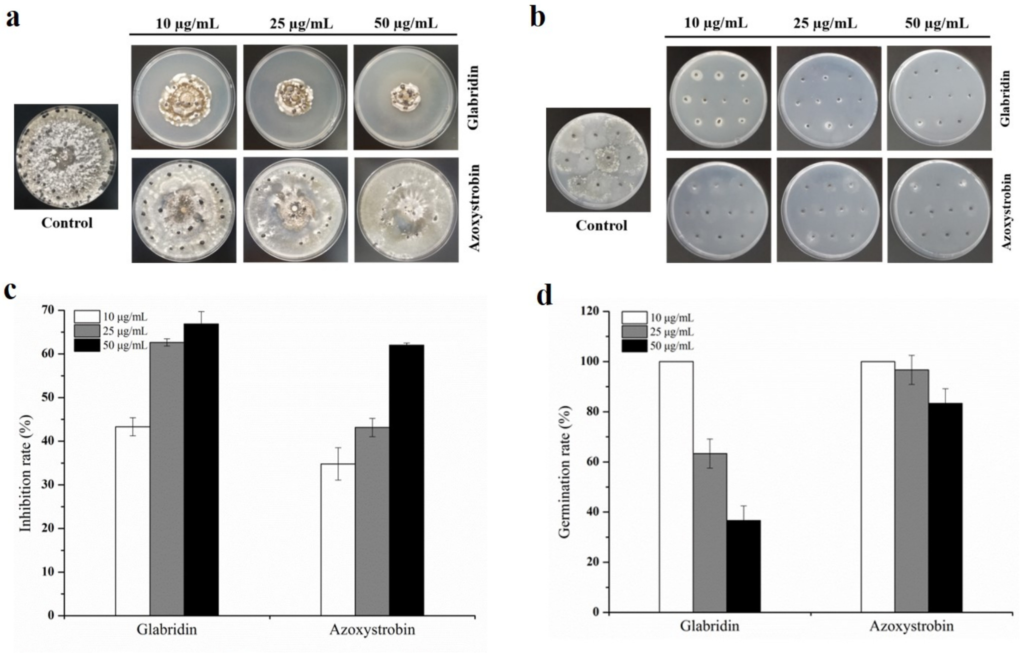

Figure S4 Effect of glabridin on sclerotia formation and germination of S. sclerotiorum.
